# Systems Toxicology Approach to Identifying Paracetamol Overdose

**DOI:** 10.1002/psp4.12298

**Published:** 2018-04-18

**Authors:** Chantelle L. Mason, Joseph Leedale, Sotiris Tasoulis, Ian Jarman, Daniel J. Antoine, Steven D. Webb

**Affiliations:** ^1^ Department of Applied Mathematics Liverpool John Moores University Liverpool UK; ^2^ EPSRC Liverpool Centre for Mathematics in Healthcare, Department of Mathematical Sciences University of Liverpool Liverpool UK; ^3^ MRC Centre for Inflammation Research Queens Medical Research Institute, University of Edinburgh Edinburgh UK

## Abstract

Paracetamol (acetaminophen (APAP)) is one of the most commonly used analgesics in the United Kingdom and the United States. However, exceeding the maximum recommended dose can cause serious liver injury and even death. Promising APAP toxicity biomarkers are thought to add value to those used currently and clarification of the functional relationships between these biomarkers and liver injury would aid clinical implementation of an improved APAP toxicity identification framework. The framework currently used to define an APAP overdose is highly dependent upon time since ingestion and initial dose; information that is often highly unpredictable. A pharmacokinetic/pharmacodynamic (PK/PD) APAP model has been built in order to understand the relationships between a panel of biomarkers and APAP dose. Visualization and statistical tools have been used to predict initial APAP dose and time since administration. Additionally, logistic regression analysis has been applied to histology data to provide a prediction of the probability of liver injury.

**Study Highlights**

**WHAT IS THE CURRENT KNOWLEDGE ON THE TOPIC?**

☑ The current clinical framework for predicting paracetamol overdose is imprecise, predominantly due to a dependency on uncertain information from patients, such as dose amount and time since administration.

**WHAT QUESTION DID THIS STUDY ADDRESS?**

☑ Mathematical modeling and statistical methods are applied to predict dose, time‐since‐administration, and the probability of paracetamol‐induced liver injury based on biomarker information by exploiting the relative abundance and quality of mouse data.

**WHAT DOES THIS STUDY ADD TO OUR KNOWLEDGE?**

☑ A new *in silico* paracetamol toxicity identification framework is described to simulate the PK/PD behavior of paracetamol and a panel of corresponding toxicity biomarkers with considerable translational potential.

**HOW MIGHT THIS CHANGE DRUG DISCOVERY, DEVELOPMENT, AND/OR THERAPEUTICS?**

☑ Systems toxicology approaches to direct biomarker identification and optimization can also be used to develop predictive modeling frameworks for other hepatotoxic drugs. An understanding of complex biological system interactions is required to refine potential treatment strategies and improve safety, ethics, and cost‐efficiency. Mathematical modeling provides an enhanced mechanistic understanding, whereas statistical modeling can provide robust, physiologically relevant predictions to underpin future investigations.

Paracetamol (acetaminophen (APAP)) is the most commonly used painkiller in the world^1^ and the leading cause for acute liver failure in the Western world.^2^ The current antidote used to treat cases of APAP overdose, N‐acetylcysteine (NAC), reduces the likelihood of progression into drug‐induced liver injury (DILI).^3^ NAC is highly effective when administered within 8–10 hours of initial APAP dose.^4^ Although NAC is currently the most effective APAP overdose treatment, there are many adverse side effects, such as rash, vomiting, and anaphylactoid reaction.^3^ The decision to administer NAC is currently based upon the nomogram treatment line,^5^ which is influenced by a measurement of alanine aminotransferase (ALT), but is also heavily dependent on the initial dose amount and time elapsed since ingestion,^6^ information that is often highly unpredictable within the clinical setting.

ALT elevation represents probable liver injury postoccurrence^7^ and is the most widely used blood‐based biomarker for measuring DILI.^8^ Aspartate aminotransferase (AST) is another DILI biomarker^8^ that accumulates in the blood due to liver damage, but it is also linked to other pathologies (e.g., heart injury).^9^ Increased serum total bilirubin is indicative of the substantial loss of functional hepatocytes; therefore, similar to ALT, this biomarker does not predict hepatotoxicity potential but instead is a postoccurrence indicator.^7^ In order to improve the treatment of APAP‐induced DILI via NAC therapy, biomarkers are required that can predict liver damage *a priori*. Although there are clear limitations, clinics currently analyze changes in ALT, AST, and total bilirubin in combination to predict DILI.^10^ Recently, biomarkers K18 and HMGB1 have been shown to add value to the measurement of ALT^11^ and have the potential to predict DILI pre‐occurrence. However, such new biomarkers are often examined singly and clarification of their functional relationships is required to aid clinical implementation.^12^ For a thorough review of the mechanisms of DILI see, for example, ref. 13.

*In silico* modeling allows for the development of mechanistic understanding of biological systems, which may not always be possible from *in vitro/in vivo* experiments alone. An interdisciplinary, systems toxicology approach is a cost‐effective way of understanding and predicting drug efficacy and toxicology while complying with the 3R's (scientific framework for use of animals in research).^14^ There have been multiple *in silico* models that have been previously developed to study APAP metabolism and associated toxic potential. Reith *et al*.^15^ produced a system of equations with parameters fitted to human data consisting of patients dosed with pain relievers to provide clarification of the role of the glucuronidation and sulfation pathways, providing a basis for examining APAP metabolism in various disease states. Diaz Ochoa *et al*.^16^ took a multiscale approach by first creating a spatiotemporal prediction of drug and metabolite concentrations within the liver, and then at the whole‐body level, including blood‐flow between organs. Remien *et al*.^17^ created a model for acetaminophen‐induced liver damage and derived ordinary differential equations (ODEs) describing changes in AST, ALT, and INR. The authors optimized initial APAP dose amount and time since overdose by fitting the resulting ODEs to clinical data (from 53 patients who overdosed). Remien *et al*.^18^ then extended this framework to a cell‐based model. Our study extends Remien's approach by combining ALT with additional biomarkers that have the potential to predict APAP‐induced liver injury pre‐occurrence. Additionally, the study is extended to nonoverdose and overdose cases in an attempt to identify the key biomarkers that discriminate between the two situations. Ben‐Shachar *et al*.^19^ created a retrospective study complementary to Remien's model. Although Remien's model aimed to predict overdose occurrence, Ben‐Shachar's model was used to determine whether an overdose would lead to fatal liver damage. Reddyhoff *et al*.^20^ constructed a cell‐based model that described major pathways impacting on APAP clearance. Sensitivity analysis determined which parameters had the largest effect on the progression to toxicity. Shoda *et al*.^21^ mechanistically modeled the biomarker HMGB1. Their focus was the role of HMGB1 with regard to the innate immune response and concluded that HMGB1 was a key input for immune cell activation.

In this report, our focus is to investigate HMBG1 within a panel of DILI biomarkers, attempting to predict APAP toxicity in mice. We propose a novel framework to predict initial APAP dose, time‐since‐administration, and the probability of APAP‐induced liver injury. The platform is distinctive primarily due to the use of promising biomarkers, optimized within the pharmacokinetic/pharmacodynamic (PK/PD) framework by combining the use of deterministic modeling with statistical analysis. The mouse is widely considered to be a good model for APAP toxicity prediction in humans^22^ and we have utilized mouse‐derived data in this study to develop our new *in silico* framework by exploiting the rich datasets available and also to avoid, at this early stage of model development, the uncertainties associated with APAP human overdose data. Translation to the human clinical case would be, in theory, a relatively simple adjustment of the PK/PD model parameters, which could be estimated from a population‐pharmacokinetic (Pop‐PK) analysis of clinical overdose data.^23^ However, the key feature of this current work is to demonstrate the development and validation of our new predictive framework using the more amenable mice data. The results from our investigation define currently undocumented pharmacokinetic (PK) parameters for APAP in mice, and the biomarkers are examined as a panel, rather than individually. Additionally, the focus of this work is the biomarkers that work well for DILI prediction due to APAP, which may only represent certain pathways or mechanisms that are not applicable with other drugs but we anticipate that this *in silico* approach can be translated across drug space with the necessary biomarker data.

## METHODS

### Model development (i) – APAP pharmacokinetics

Four datasets from two separate published studies^24^, ^25^ recording APAP concentration over time in mice following intraperitoneal administration of 50, 150, 500, and 530 mg/kg doses were used to parameterize a two‐compartment PK model describing APAP metabolism in mice. Note that for applications to oral administration, the absorption rate parameter, $k_{a}$, would be multiplied by a bioavailability fraction to implicitly take into account effects of gastric emptying and absorbed fraction (details of the model selection can be found in the **Supplementary Material**).

Two ODEs were used to represent changes in APAP concentration within two PK compartments (central and peripheral) of the mice in the following system:

(1) $$\frac{dC_{c}}{dt}=\frac{k_{a}D_{0}e^{-k_{a}t}}{V_{c}}+k_{21}C_{p}\frac{V_{p}}{V_{c}}-k_{12}C_{c}-k_{el}C_{c},$$

(2) $$\frac{dC_{p}}{dt}={\;k}_{12}C_{c}\frac{V_{c}}{V_{p}}-k_{21}C_{p},$$

where $C_{c}$ represents the central compartment concentration of APAP (μmol/l), $C_{p}$ represents the peripheral compartment concentration of APAP (μmol/l), $k_{a}$ represents the absorption rate from the peritoneal cavity (h^−1^), $D_{0}$ represents initial dose (mg), $k_{21}$ represents the transfer rate from the peripheral to the central compartment (h^−1^), $k_{12}$ represents the transfer rate from the central to the peripheral compartment (h^−1^), $V_{p}$ represents the theoretical volume of the peripheral compartment (l/kg), $V_{c}$ represents the theoretical volume of the central compartment (l/kg), $k_{el}$ represents the overall elimination rate (summation of both excretion and metabolism processes) (h^−1^), and t represents the time variable (h).

Solving both equations analytically through Laplace transforms^26^ gives the following equation for paracetamol concentration in the central compartment as a function of time:

(3) $$C_{c}\left(t\right)=\frac{k_{a}D_{0}}{V_{c}}[\frac{\left(k_{21}-\;\alpha \right)}{\left(k_{a}-\;\alpha )(\beta -\alpha \right)}e^{-\alpha t}+\frac{\left(k_{21}-\beta \right)}{{(k}_{a}-\;\beta )(\alpha -\beta )}e^{-\beta t}+\frac{\left(k_{21}-k_{a}\right)}{\left(\alpha -k_{a})(\beta -k_{a}\right)}e^{-k_{a}t}],$$

where α and β are related to the model parameters as follows:

$$\alpha =\frac{1}{2}\left(k_{12}+k_{21}+k_{el}+\sqrt{{\left(k_{12}+k_{21}+k_{el}\right)}^{2}-4k_{21}k_{el}}\right),$$

and

$$\beta =\frac{1}{2}\left(k_{12}+k_{21}+k_{el}-\sqrt{{\left(k_{12}+k_{21}+k_{el}\right)}^{2}-4k_{21}k_{el}}\right).$$

Eq. (3) was fitted to the four aforementioned datasets simultaneously using a Nelder‐Mead search algorithm,^27^ with parameters $k_{a},\;k_{21},\;V_{c},\;\alpha$, and β being optimized in order to minimize the difference between the model output and the observed APAP dynamics. Note that all subsequent data fitting also uses this algorithm. Data fitting was performed using the fminsearch tool in Matlab.^28^ Optimized parameter values and model simulation codes are provided in the **Supplementary Material**.

### Model development (ii) – glutathione depletion

The role of glutathione (GSH) in APAP metabolism is to detoxify N‐acetyl‐p‐benzoquinoeimine (NAPQI), a highly reactive metabolite^13^ formed following the bioactivation of APAP. Therefore, GSH stores are depleted in the case of an overdose and NAPQI accumulates, eventually causing liver damage. In our model, paracetamol biomarker response dynamics were assumed to be directly dependent on GSH depletion. The GSH parameter values were optimized such that the solution was fitted to GSH time‐course data from a literature study.^25^ GSH dynamics are described in the Eq. (4) below:

(4) $$\frac{d[gsh]}{dt}=k_{o}\cdot gsh_{0}-k_{o}\cdot gsh-\frac{\xi \cdot k_{el}\cdot C_{c}\cdot gsh}{gsh+k_{pr}},$$

where $k_{o}$ is the basal removal rate (including background usage) of GSH (h^−1^), $gsh_{0}$ is the baseline value of GSH (μmol/l) in the APAP‐free steady state, ξ is the proportion of eliminated APAP that is transformed into NAPQI, and $k_{pr}$ is the ratio of NAPQI forming other protein adducts relative to NAPQI detoxified by GSH. The APAP elimination rate, $k_{el}$, was identified during PK model development (above), whereas all other parameters were optimized by fitting Eq. (4) to the data in Antoine *et al*.^25^ Further information, and full derivation of the GSH ODE in Eq. (4) is described in the **Supplementary Material**.

### Model development (iii) – pharmacodynamics

The toxic response to APAP overdose was mathematically described with individual pharmacodynamic (PD) models representing biomarker concentrations ( r = ALT, HMGB1, K18, and fragmented K18) over time, as described in Eq. (5):

(5) $$\frac{dr}{dt}=r_{0}k_{out}\left(\frac{R_{50}^{n}+{gsh}_{0}^{n}}{R_{50}^{n}}\right)\left(1-\frac{{gsh}^{n}}{R_{50}^{n}+gsh^{n}}\right)-k_{out}r,$$

where $r_{0}$ is the biomarker baseline concentration, $\;k_{out}$ is the natural decay rate of the biomarker (h^−1^), $R_{50}$ represents the concentration of GSH, which causes the biomarker production (response) to be half its maximal value (μmol/l), and n is a parameter that reflects the steepness of the biomarker production term.^29^ Further model details can be found in the **Supplementary Material**. Although parameter values $r_{0}$ and ${gsh}_{0}$ can be identified directly from the data, $k_{out},\;R_{50},$ and n were optimized by individually fitting the model output to data measuring biomarker concentration over time following a 530 mg/kg dose of APAP.^25^

### Model validation

The parameterized PK/PD model was validated against data from a separate experiment (detailed below). The PK/PD model simulated several dosing scenarios 0, 150, 300, and 530 mg/kg and biomarker concentration outputs were extracted at 5 hours and compared with the experimental data. Further details and results can be found in the **Supplementary Figure S1**.

### Experimental animal treatment

The protocols described were undertaken in accordance with criteria outlined in a license granted under the Animals (Scientific Procedures) Act 1986 and approved by the University of Liverpool Animal Ethics Committee. Groups of six individual CD‐1 male mice (25–35 g) with free access to food and water were included in the study. For the biomarker time‐course, treatment was as previously described.^25^ For the dose/response data used for validation, study animals were administered a 150, 300, or 530 mg/kg i.p. APAP injection and were euthanized 5 hours post‐treatment. The 5‐hour time point has been used in previous studies,^25^ and was chosen here not only because the pathological and biomarker response has been extensively categorized at this point, but the majority of key mechanisms (apoptosis, necrosis, and inflammation) are also identifiable at this time point. Control animals received either 0.9% saline or solvent control in 0.9% saline as appropriate. Serum ALT activity, HMGB1, and fragmented K18 levels were determined, and GSH content assessment was carried out on the livers of all animals. Total hepatic glutathione (GSH and oxidized glutathione) levels and biomarker quantification/characterization were determined as described previously.^25^, ^30^

## PREDICTING TIME SINCE ADMINISTRATION AND INITIAL DOSE

### Multiple linear regression

The *in silico* model was used to create virtual datasets for testing and validation (**Methodology in Supplementary Material**). A robust multiple linear regression model^31^ was fitted to the *in silico* derived data to predict time‐since‐administration and initial dose.

### Visualization

Principal component analysis (PCA),^32^ and the T‐SNE method^33^ were applied to visualize the simulated *in silico* datasets with regard to linear combinations of all variables (APAP and toxicity biomarkers combined) for each *in silico* individual.

### Classification

Appropriate classes for each dose and time range were identified to see if the time‐since‐administration and dose amount could be predicted for a new individual within the population. Various classification techniques (detailed in **Supplementary Material**) appropriate for such a task were used and compared.

### Predicting probability of liver injury

The biomarker time‐course experimental data used to create the PD model^25^ also provided a corresponding histology score for each mouse from the range 0, 1, 2, and 3. These histology scores were binarized based upon previously published criteria.^25^ Forward‐stepwise binary logistic regression^34^ was applied in order to understand the most significant biomarker or panel of biomarkers for DILI. The most significant biomarkers were then used in combination with PK/PD model simulations to predict the DILI probability.^35^

Further details of all aforementioned statistical techniques can be found in the **Supplementary Material**.

## RESULTS

**Figure 1:**
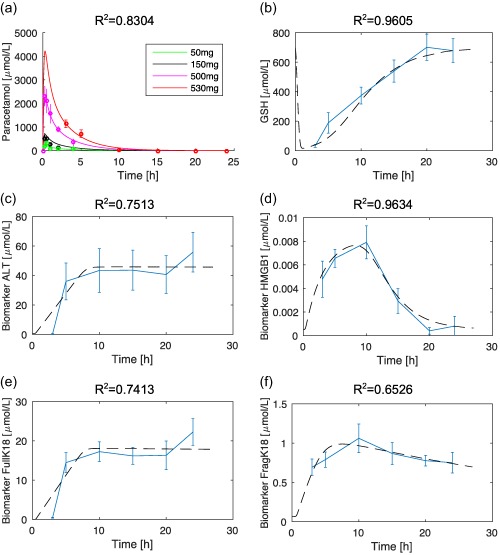
*In silico* simulation outputs from the optimized model compared with the experimental data. (**a**) Acetaminophen (APAP) pharmacokinetic simulations (solid lines) comparable to original data values with green, black, magenta, and red representing APAP time‐course following a 50, 150, 500, and 530 mg/kg dose, respectively. (**b**) Glutathione (GSH) simulations (black dashed lines) comparable to original data (blue). Individual pharmacodynamic simulation (black dashed lines) comparable to data (blue) for biomarkers alanine aminotransferase (ALT) (**c**), HMGB1 (**d**), full K18 (**e**), and fragmented K18 (**f**).

Results from the parameter optimization of the PK/PD models can be seen in **Figure** 1. Note that sufficient early time experimental APAP plasma concentrations are currently unavailable, which would verify the accuracy of time of maximum plasma concentration (T_max_) and peak plasma concentration (C_max_) of the 530‐mg dose. Nevertheless, with an *R^2^* value of 0.8304 for the PK model, and values of 0.7513, 0.9634, 0.7413, and 0.6526 for the PD models for ALT, HMGB1, K18, and fragmented K18, respectively, it is shown that the *in silico* model recapitulates *in vivo* experimental dynamics. Optimized parameters for all of the PK/PD models can be found in the **Supplementary Table S1**.

The $R_{50}$ parameter in the biomarker PD models defines a concentration of GSH at which the biomarker has reached half of its maximal production rate (MPR). For biomarkers ALT, HMGB1, K18, and fragmented K18, the $R_{50}$ values were 227.67, 399.08, 212.87, and 72.09 μmol/L, respectively. Therefore, in the model, as GSH is depleted from a baseline of 696.91 μmol/L^36^ and reaches a concentration 399.08 μmol/L (42.73% depletion), HMGB1 has reached half of its MPR and is, therefore, considered to be the fastest responding biomarker. GSH must be further depleted to 227.67 μmol/L and 212.87 μmol/L (67–69% depletion), respectively, before biomarkers ALT and K18 reach half of their MPR. Approximately 90% GSH depletion is required for fragmented K18 to reach half of its MPR in the model.

### Identifying time/dose category following APAP dose

**Figure 2:**
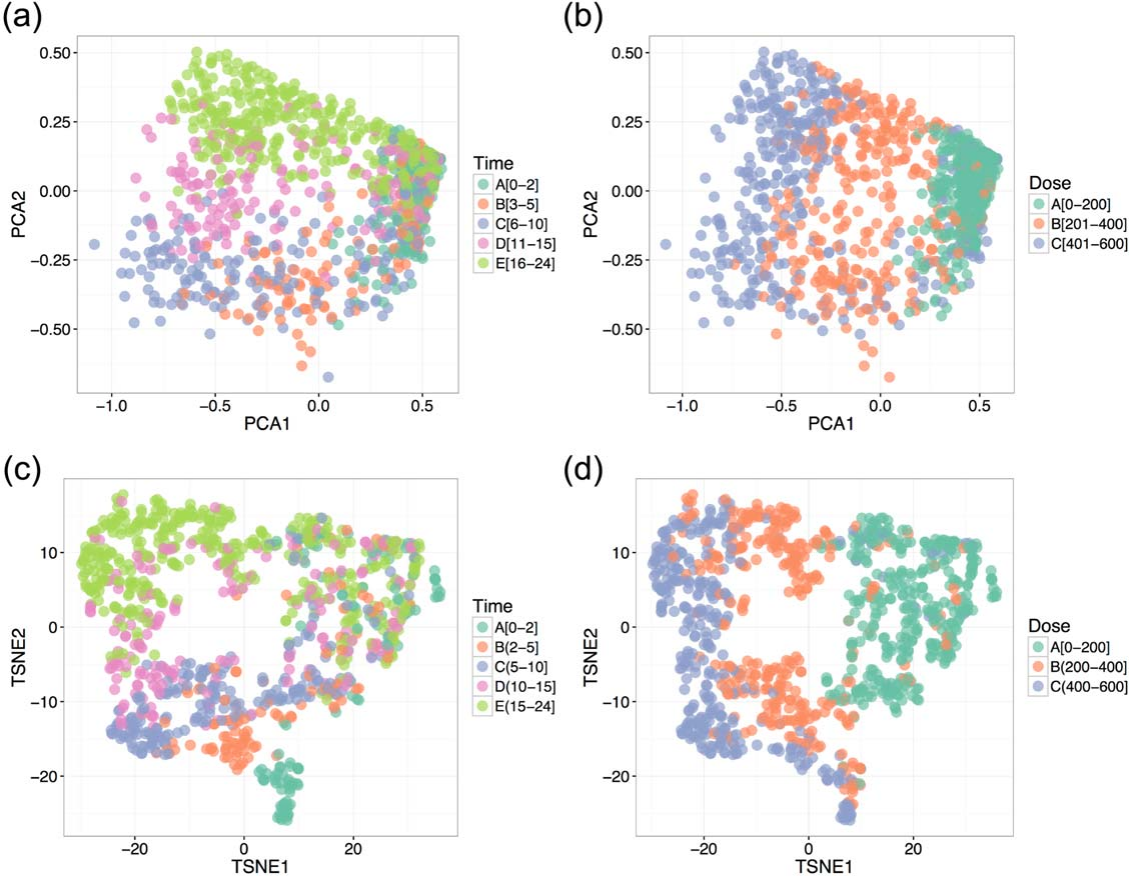
Visualization and classification of time‐since‐administration and dose results. For time‐since‐administration, dark green represents class 0–2, orange represents 2–5, blue represents 5–10, pink represents 10–15, and pale green represents 15–24 hours. For dose, green represents 0–200, orange represents 201–400, and blue represents 401–600 mg/kg. (**a, b**) Two‐dimensional Principal Components Analysis (PCA) visualization of *in silico* mouse observations with respect to time‐since‐administration and dose, respectively. (**c, d**) Two‐dimensional TSNE visualization of *in silico* mouse observations with respect to time‐since‐administration and dose, respectively.

Projecting the *in silico* derived data onto the principal components and visualizing with respect to time‐since‐administration and dose amount, as can be seen in **Figure** 2 **a,b**, allowed classes to be clearly distinguished with minimal level of overlap confirming the biomarker utility in class prediction. The level of class overlap with respect to dose is significantly lower. Visualizing the data with the T‐SNE method (**Figure** 2 **c‐d**) enhances the previous visualization, and that dose may be separated more accurately. Additionally, the time‐since‐administration classes are more separable with the T‐SNE method, particularly with earlier time ranges.

The classification results are consistent across the different methodologies (**Table** 1). Should a new observation arise, this framework could predict which “time‐since‐administration” and “dose” category it should be placed in with 73.7% and 86.5% accuracies, respectively. The results of the linear regression model used to evaluate time‐since‐administration and initial dose, both as continuous variables, are reported in **Table** 2. In both cases, the model is significant at the 99% confidence level. The *R^2^* values indicate that when predicting time‐since‐administration, ∼53% of the variance in results can be explained by the model, whereas when predicting dose ∼80% of the variation can be explained by the model. An exact time‐since‐administration value was able to be predicted with a residual standard error and accuracy of 3.6 hours, whereas an exact dose was predicted with only an error of 56.81 mg/kg.

**Table 1:** Classification results for several algorithms with respect to time‐since‐administration and dose, respectively, with numbers representing levels of accuracy

Classification method	Time accuracy	Dose accuracy
Multinomial logistic regression	0.728	0.865
Ordinal multinomial logistic regression	0.570	0.859
Naive Bayes	0.689	0.844
Linear discriminant analysis	0.657	0.860
Quadratic discriminant analysis	0.737	0.853
K‐nearest neighbor	0.664	0.859
Optimal weighted nearest neighbor	0.676	0.858

For example, the multinomial logistic regression model can predict time‐since‐administration with 72.8% accuracy.

**Table 2:** Multiple linear regression analysis results ‐ summary statistics for models used to predict both time‐since‐administration and dose

	Dependent variable (coefficient and related error)	
	Time (1)	Dose (2)
APAP concentration	‐18.141*^**^ (1.095)	445.602^***^ (13.865)
ALT concentration	2.402^**^ (0.988)	94.724^***^ (12.830)
HMGB1 concentration	‐15.928^***^ (0.636)	
Full K18 concentration	8.964^***^ (0.837)	241.527^***^ (12.958)
Fragmented K18 concentration		310.574^***^ (13.260)
Constant	14.812^***^ (0.268)	67.068^***^ (3.193)
Observations Residual SE (df = 994)	1,000 3.593	1,000 56.805

The first number in each element of the table represents the biomarker coefficient in the regression model, whereas the second number represents the coefficient's corresponding error. For example, −18.141 is the APAP concentration coefficient in the model predicting time‐since‐administration, and this coefficient has an error of 1.095. The significance of each biomarker in the model is indicated by the number of asterisks ^*^ *P* < 0.1; ^**^ *P* < 0.05; ^***^ *P* < 0.01.

ALT, alanine aminotransferase; APAP, acetaminophen.

### Predicting the probability of liver injury following an APAP dose

**Figure 3:**
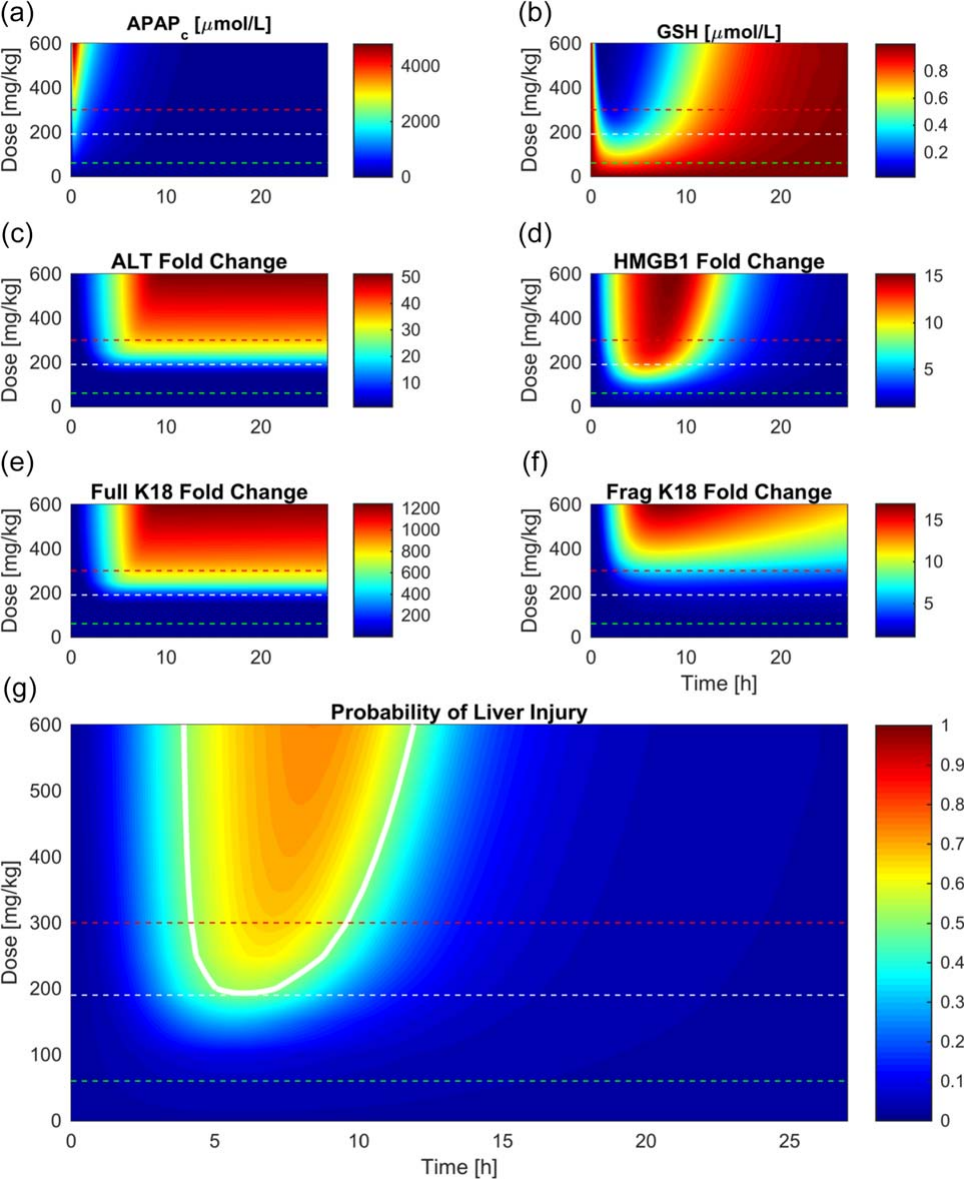
(**a–f**) Fold‐changes in biomarker concentration relative to their baseline values over time 0–24 hours for acetaminophen (APAP), glutathione (GSH), alanine aminotransferase (ALT), HMGB1, full K18, and fragmented K18, respectively, following APAP doses ranging from 0–600 mg/kg. (**g**) Proposed framework for predicting probability of liver injury dependent upon dose, time, and HMGB1 concentration. The white contour indicates the threshold of probability 0.5 of liver injury, the red dashed‐line represents currently used APAP dose for toxicity studies in mice, the white dashed‐line represents toxic dose proposed by our model, and the green dashed‐line indicates current known therapeutic dose for mice.

From the forward‐stepwise logistic regression analysis, the model that used HMGB1 concentration alone as a predictor had the highest significance (*P* value 0.003). **Figure** 3 **a‐f** represents the fold‐changes in biomarker concentrations with respect to time following various doses. For higher doses, APAP and related toxicity biomarker concentrations are significantly increased during the time course, whereas GSH is significantly decreased at higher doses, representing depletion of stores. **Figure** 3 **g** shows how the probability of serious liver injury (dependent only on HMGB1 concentration as predicted by the logistic regression model) changes over time for doses between 0 and 600 mg/kg. A threshold probability of 0.5 (i.e., 50% liver injury likelihood) was used to determine likeliness of DILI. Any observation within the white contour boundary is, therefore, predicted likely to be a concentration representative of liver injury (i.e., 50% chance). For lower toxic doses, according to the model, HMGB1 concentrations that likely indicate liver injury are most apparent between 5 and 10 hours postdose. As the dose increases, the time frame increases to ∼5–15 hours. Note that combinations of APAP/ALT and APAP/full K18 were also significant; therefore, these biomarker combinations could be investigated in the case of predicting DILI following late presentation of paracetamol toxicity and prognosis within the 24‐hour window.

Currently, toxicity is thought to be apparent in mice after a 300 mg/kg dose, shown by the red line in **Figure** 3 **g**. Our binary logistic regression (model based solely on HMGB1 concentration) states there is >50% chance of liver injury at a 200 mg/kg dose, shown by the white contour in **Figure** 3 **g**. The currently used toxic dose (300 mg/kg) coincides with around 90% GSH depletion, which can be seen in **Figure** 3 **b**. This coincides with a relationship well known in the literature.^13^ This toxic level is also the dose at which fragmented K18 begins to elevate, as shown in **Figure** 3 **f**. The toxic dose proposed by the *in silico* model (200 mg/kg) is the dose at which ALT and full K18 begin to elevate (**Figure** 3 **c** and **Figure** 3 **e**, respectively) and HMGB1 first reaches peak concentration (**Figure** 3 **d**).

### Visualizing the probability of liver injury following an APAP dose

**Figure 4:**
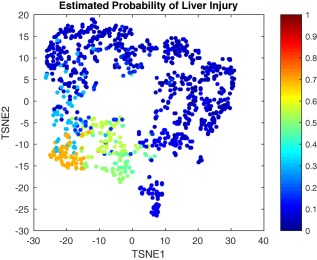
Two‐dimensional TSNE visualization of *in silico* mouse observations with respect to estimated probability of liver injury.

Combining the PCA/T‐SNE analysis with our proposed framework for predicting the probability of liver injury allowed the virtual datasets to be visualized not only with regard to the initial dose and time since ingestion, but also the subsequent probability of liver injury. With reference to **Figure** 4, observations with a high probability of liver injury are clearly clustered within the parameter space and separable from low probability cases. Additional similar projections (with both the PCA and T‐SNE methods), including the estimated maximum probability of liver injury for each observation, are shown in the **Supplementary Material**.

## DISCUSSION

The current clinical framework for predicting whether or not APAP antidote treatment is necessary is highly dependent upon information provided by the patient, such as when the dose was taken and in what quantity. This information is often vague and/or unreliable. Consequently, critically vulnerable patients are often left untreated or, conversely, NAC is unnecessarily administered. Changes in legislation have already led to an estimated increased cost of £8.3 million per year due to overused NAC treatment.^37^ Mathematical and statistical analysis provide a proof‐of‐concept tool to predict information with a much higher level of certainty, based on a panel of promising biomarkers.

We have developed an optimized PK/PD model for APAP and appropriate biomarkers of liver injury in a systems toxicology approach. The model was used to conduct investigations within a dosing range of 0–600 mg/kg without any further *in vivo* testing. The optimized *in silico* framework is suitable for use in further theoretical investigations, providing a greater scope for reducing the dependency on animal testing in toxicity and complying with the 3Rs principles.^14^ For example, results from our analysis could improve experimental refinement, such as predicting the probability of liver injury and toxicity at 200 mg/kg in mice rather than 300 mg/kg. Not only may experimentalists be dosing mice at amounts higher than necessary, they may also be missing vital information apparent at lower doses.

APAP‐induced liver toxicity is thought to occur when GSH depletes by around 80–90%,^13^ which coincided with elevated fragmented K18 levels. The *in silico* PD model and its reported *R_50_* values suggest that levels of HMGB1, ALT, and full K18 elevate prior to this depletion level, elevating at 43%, 67%, and 69%, respectively. As a result, HMGB1 in particular could be considered as an earlier indicator of DILI.

The identification of more accurate predictions of dose timing and amount, informed by biomarker concentration samples, will improve nomogram treatment line accuracy.^6^ Predictions for the time‐since‐administration were successfully categorized into 0–2, 2–5, 5–10, 10–15, and 15–24‐hour ranges based on APAP, ALT, HMGB1, and full K18 concentration values with 73.7% accuracy. Should this framework be translated to a similar level of efficiency in the human clinical case, this information will have impact regarding the determination of the potential liver injury, with less dependency on patient information. Additionally, an exact value was predicted with an accuracy of 3.6 hours. Similarly, initial dose was able to be classified into 0–200, 201–400, and 401–600 mg/kg categories with 86.5% accuracy and an exact dose predicted with an expected error of ± 56.81 mg/kg. A panel of biomarker measurements could be used in this manner to provide the dose and time information, which will identify a (time‐dose) point on the liver injury framework, provided in **Figure** 3 **g**, from which one can read off an instantaneous probability of liver injury and how this probability is predicted to change as time progresses. Obtaining dose and time information based on biomarker concentrations and combining this with our proposed liver injury framework shows the utility of these biomarkers in predicting dose amount, time since ingestion, and the subsequent probability of liver injury.

Although ALT concentration is currently used as a clinical measure to inform potential toxicity, it was found to have the least importance in the regression model for predicting time‐since‐administration as a continuous variable. Out of all the biomarkers used in the multiple linear regression analysis, HMGB1 was found to have the highest time‐since‐administration model coefficient. This analysis suggests, therefore, that not only is HMGB1 an earlier indicator of DILI, but it is also an important biomarker in accurately predicting the time elapsed since administration. Furthermore, logistic regression analysis identified HMGB1 as the most significant predictor for liver injury, in line with recent studies defining HMGB1 as a more sensitive DILI predictor.^38^ As noted above, the focus of this work has been the biomarkers that work well for DILI prediction due to APAP, in which case HMGB1 is highlighted by our analysis. However, for different drugs, DILI may involve different mechanisms and, as such, HMGB1 may not perform so well as a singular biomarker but instead a panel would be more predictive.

Although the results from the T‐SNE method for visualization showed clear separation, particularly with regard to the probability of liver injury, there was a slight overlap in the time‐since‐administration and dose plots. This result supports the possibility of defining further classes through unsupervised methodologies in future investigations. The classification techniques used provided incredibly high accuracy levels considering the nature of the problem. A further investigation of interest is the rate of misclassification between the classes with regard to critical errors at the edges of the variable ranges.

The framework proposed has the potential for substantial clinical impact once translated to human. The analysis was applied to mice due to the relative abundance and quality of data (especially for toxicity cases) and the quantity of relevant biomarker data required to properly characterize such a mathematical and statistical predictive framework. Equivalent APAP clinical data is available but has a tendency to be noisy, sparse, and inconsistent. Analysis of such data would, therefore, require the significant application of (top‐down) Pop‐PK to unravel the stochasticity of the mixed‐effects involved, in addition to understanding and capturing the mechanisms of the PK/PD problem. For example, the relative influence of variation in certain model parameters on quantitative model outputs can be determined by sensitivity analysis, allowing for identification of mechanistic processes that would require particularly careful consideration when translating this model to a human clinical Pop‐PK framework (see **Supplementary Materials** for further details).

An advantage of our study is that the same biomarkers can be measured in both humans and animals by the same methodologies. Moreover, the model hepatotoxin we have used, acetaminophen, is directly comparable between human and mice with respect to mechanism of toxicity and action of the antidote. The major differences between human and mouse studies are the mass dose of acetaminophen needed to induce toxicity in mice and the kinetics of the biomarker profile.^25^, ^39^, ^40^ The dose response in mice is well documented and is consistent with our data. Furthermore, this can be adjusted as a parameter within our model to reflect the clinical situation. There are a number of clinical studies now published that have measured these biomarkers from human studies in a time‐dependent way.^41^, ^42^ The approach we describe to modify dose adjustment can also be undertaken to reflect biomarker kinetic differences. It is important to note that it is difficult to properly obtain or assess human pathology in the acute setting, and it is only really in the event of liver transplantation that we see a strong relationship between humans and mice.^25^, ^43^ Given the strong relationship between the biomarker signatures and mechanism of APAP action between humans and mice, it would be reasonable to translate findings from mouse acute data,^25^, ^39^ to human acute data.^11^ Taking these points into consideration, in its current form, our framework is highly predictive and provides promise for clinical use in discriminating time‐since‐administration, initial dose amount, and subsequent probability of liver injury. This would be a significant application and could instruct the determination of NAC intervention in patients suspected of APAP overdose.

Clinical assessment of DILI is, in practice, often based on causality assessment, with expert opinion being the gold standard, and does not wholly depend on simple biochemical tests. We have recently discussed the potential improvement to laboratory‐based measures in aiding DILI assessment and one key feature we propose is that laboratory measurements should be repeated when DILI is suspected.^11^, ^44^ This could allow for the determination of the cause of injury as well as the derivation of the area under the curve (AUC) of a liver toxicity marker. A limitation of our current *in silico* model framework is that it is focused on whether or not liver injury occurs, rather than prediction of the maximum damage observed in an individual. The cause of this limitation is the sparsity of the histology data used for model parameterization. However, if such additional AUC‐based measurements could be obtained, then this could potentially offer vital data to extend the predictive potential of our *in silico* platform by quantifying the maximal liver injury and further aiding DILI assessment.

## Source of Funding

C.L.M. acknowledges funding support from the Faculty of Engineering and Technology, Liverpool John Moores University. J.L. acknowledges funding support from the Liverpool Centre for Mathematics in Healthcare (EPSRC grant EP/N014499/1).

## Conflict of Interest

The authors declared no competing interests for this work.

## Author Contributions

C.L.M., J.L., and S.D.W. wrote the manuscript. D.J.A. and S.D.W. designed the research. C.L.M., J.L., and S.D.W. performed the research. C.L.M., S.T., and I.J. analyzed the data. Daniel J. Antoine and Steven D. Webb contributed to this work equally.

## Supporting information

Supplementary Material

Model Code
