# Supplementary material for: Systems Toxicology Approach to Identifying Paracetamol Overdose
Source: CPT Pharmacometrics Syst Pharmacol. 2018 Apr 18;7(6):394–403. doi: 10.1002/psp4.12298 (PMC6027737; doi:10.1002/psp4.12298)
Supplement: Supplementary file 1 — Supplementary Material [file PSP4-7-394-s001.docx]

**Supplementary Data Description**

**Section 1** of the supplementary data defines all parameters for the mathematical models used in the analysis. Parameters values with their corresponding standard error estimates are provided, along with details on whether they were fixed/optimised during the modelling. Standard errors were computed in the Mathworks’ Simbiology toolbox using the fminsearch optimiser (1). A short description of each parameter is provided, as well as a reference to the experimental data used in optimisation. **Section 2** provides a justification of how the mathematical models were formulated, alongside a detailed derivation of the GSH equation. **Section 3** details how we validated the *in-silico* model, and provides the corresponding results of that analysis. **Section 4** provides additional information regarding the statistical analysis including: details of how the *in-silico* observations were derived, justification for the model predicting ‘time since administration’ and ‘initial dose’ as continuous variables, a short description of how the observations were visualised, justification for the choice of dose and time ranges with subsequent justification of each of the classification methods used, as well as information on how histology scores were binarised for the logistic regression analysis and finally the formulae produced from the output when aiming to predict the probability of liver injury. **Section 5** shows projections of the *in-silico* derived observations separated with respect to their estimated probability of liver injury. **Section 6** provides a sensitivity analysis of the *in-silico* model parameters.

**Supplementary Data**

1. **Model Parameter Values**

**Table S1:** Full list of parameters and corresponding standard error estimates defined for all mathematical models used in the analysis.

| Model | Parameter | Optimised? | Value | Standard Error | Parameter Description | Optimising  Dataset |
| --- | --- | --- | --- | --- | --- | --- |
| PK | $k_{a}$ (h^-1^) | Optimised | 9.05 | 1.8511 | Absorption rate from peritoneal cavity | (2,3) |
|  | $k_{el}$ (h^-1^) | Optimised | 0.52 | 0.0420 | Total elimination rate |  |
|  | $k_{12}$ (h^-1^) | Optimised | 0.42 | 0.0006 | Distribution rate from central to peripheral compartment |  |
|  | $V_{c}$ (l/kg) | Optimised | 0.02 | 0.0010 | Theoretical volume of central compartment |  |
|  | $V_{P}$ (l/kg) | Optimised | 0.01 | Fixed | Theoretical volume of peripheral compartment |  |
|  | $k_{21}$ (h^-1^) | Optimised | 1.01 | 0.4449 | Distribution rate from central to peripheral compartment |  |
| PD- GSH | ${gsh}_{0}$ | Fixed | 696.9136 |  | Baseline value of GSH (4) | (3) |
|  | $k_{pr}$ | Optimised | 71.06 | 2848.6441 | ratio of NAPQI forming other protein adducts relative to detoxification |  |
|  | $\xi$ | Optimised | 0.68 | 0.1726 | proportion of CYP activated APAP that is transformed into NAPQI |  |
|  | $k_{o}$ (h^-1^) | Optimised | 0.25 | 2.3196 | Natural decay/background usage rate of GSH |  |
|  | $k_{el}$ (h^-1^) | Fixed from previous optimiser | 0.52 |  | Total elimination rate |  |
| PD-ALT | $R_{0}$ (μmol/l) | Fixed | 0.7621 |  | Baseline value of ALT | (3) |
|  | $n$ | Optimised | 9.26 | 1.7422 | reflects the steepness of the biomarker production term |  |
|  | ${gsh}_{0}$ (μmol/l) | Fixed | 696.9136 |  | Baseline value of GSH |  |
|  | $R_{50}$ (μmol/l) | Optimised | 227.67 | 14.0245 | Concentration of GSH which causes ALT concentration to be half its maximum value |  |
|  | $k_{out}$ (h^-1^) | Optimised | 0.0002 | <0.0001 | Natural decay/background usage of ALT |  |
| PD-HMGB1 | $R_{0}$ (μmol/l) | Fixed | 0.0005 |  | Baseline value of HMGB1 | (3) |
|  | $n$ | Optimised | 4.90 | 0.1586 | reflects the steepness of the biomarker production term |  |
|  | ${gsh}_{0}$ (μmol/l) | Fixed | 696.9136 |  | Baseline value of GSH |  |
|  | $R_{50}$ (μmol/l) | Optimised | 399.08 | 24.8957 | Concentration of GSH which causes HMGB1 concentration to be half its maximum value |  |
|  | $k_{out}$ (h^-1^) | Optimised | 0.35 | 0.1586 | Natural decay/background usage of HMGB1 |  |
| PD-Full K18 | $R_{0}$ (μmol/l) | Fixed | 0.0146 |  | Baseline value of full K18 | (3) |
|  | $n$ | Optimised | 10.42 | 0.0043 | reflects the steepness of the biomarker production term |  |
|  | ${gsh}_{0}$ (μmol/l) | Fixed | 696.9136 |  | Baseline value of GSH |  |
|  | $R_{50}$ (μmol/l) | Optimised | 212.87 | 0.1225 | Concentration of GSH which causes Full K18 concentration to be half its maximum value |  |
|  | $k_{out}$ (h^-1^) | Optimised | 0.0007 | 0.1225 | Natural decay/background usage of Full K18 |  |
| PD-Fragmented K18 | $R_{0}$ (μmol/l) | Fixed | 0.0642 |  | Baseline value of Fragmented K18 | (3) |
|  | $n$ | Optimised | 2.30 | 0.0146 | reflects the steepness of the biomarker production term |  |
|  | ${gsh}_{0}$ (μmol/l) | Fixed | 696.9136 |  | Baseline value of GSH |  |
|  | $R_{50}$ (μmol/l) | Optimised | 72.09 | 0.4032 | Concentration of GSH which causes Fragmented K18 concentration to be half its maximum value |  |
|  | $k_{out}$ (h^-1^) | Optimised | 0.02 | 0.0117 | Natural decay/background usage of Fragmented K18 |  |

1. **Model Formulation**

***2.1. APAP pharmacokinetic model formulation***

Due to the biphasic response observed in the log data, we assume that instantaneous equilibrium is not achieved between the tissues of the body following administration. Therefore, we constructed a PK model describing distribution and elimination from two distinct compartments representing central (highly perfused) and peripheral (poorly perfused) tissues following rapid absorption (5). Two-compartment models are widely used in APAP PK modelling, for example see (39, 40). Subsequently, two ordinary differential equations (ODEs) were used to represent changes in APAP concentration in both PK compartments of the mice in the following system:

|  | $\frac{dC_{c}}{dt}=\frac{k_{a}D_{0}e^{-k_{a}t}}{V_{c}}+k_{21}C_{p}\frac{V_{p}}{V_{c}}-k_{12}C_{c}-k_{el}C_{c},$ | (S1) |
| --- | --- | --- |
|  | $\frac{dC_{p}}{dt}=k_{12}C_{c}\frac{V_{c}}{V_{p}}-k_{21}C_{p}.$ | (S2) |

***2.2. GSH ODE formulation***

APAP is metabolised predominantly by the phase II pathway via glucuronidation and sulphation but at high doses, the sulphation process saturates and an increased amount of APAP is metabolised by the phase I (CYP) pathway. In this case, APAP combines with cytochrome P450 to create N-acetyl-p-benzoquinoeimine (NAPQI), a highly toxic metabolite (8). NAPQI can be detoxified by glutathione (GSH). However, with large doses of APAP, GSH stores deplete (9) and NAPQI accumulates leading to the possibility of DILI. It was for this reason that the biomarker response dynamics were modelled to be dependent on GSH depletion.

To formulate the rate of change of GSH, the following mass-action-based ODE was initially considered:

| $\frac{d\left[ gsh \right]}{dt}=k_{i}-k_{o}\left[ gsh \right]-k_{G}\left[ NAPQI \right]\left[ gsh \right].$ | (S3) |
| --- | --- |

Where $k_{i}$ (h^-1^) is the background/natural production rate of GSH, $\left[ gsh \right]$ (μmol/l) is the concentration of GSH, $k_{o}$ (h^-1^) is the natural decay/background-usage rate of GSH and $k_{G}$ (h^-1^$\mu M^{-1}$) is the decay rate of GSH due to binding with NAPQI. The model was simplified by assuming the rate of change of NAPQI as follows,

| $\frac{d\left[ NAPQI \right]}{dt}=\xi k_{el}\left[ C_{c} \right]-k_{G}\left[ NAPQI \right]\left[ gsh \right]-k_{p}\left[ NAPQI \right],$ | (S4) |
| --- | --- |

where $\xi$ is the proportion of CYP-activated APAP that is transformed into NAPQI, $k_{el}$ (h^-1^) is the total rate of APAP elimination, and $k_{p}$ (h^-1^) is the rate at which NAPQI binds to other (non-GSH) proteins.

Because NAPQI is short-lived and the associated reactions are rapid on the time-scale of APAP depletion, we assume $\left[ NAPQI \right]$ is at a quasi-steady state on the time-scale of interest, namely,

| $0=\xi k_{el}\left[ C_{c} \right]-k_{G}\left[ NAPQI \right]\left[ gsh \right]-k_{p}\left[ NAPQI \right],$ | (S5) |
| --- | --- |

and thus,

| $\left[ NAPQI \right]=\frac{\xi k_{el}\left[ C_{c} \right]}{k_{G}\left[ gsh \right]+k_{p}}.$ | (S6) |
| --- | --- |

Substituting this term into Equation (S3) we obtain,

| $\frac{d\left[ gsh \right]}{dt}=k_{i}-k_{o}\left[ gsh \right]-\frac{\left[ gsh \right]\xi k_{el}\left[ C_{c} \right]}{\left[ gsh \right]+k_{pr}},$ | (S7) |
| --- | --- |

where $k_{pr}= \frac{k_{p}}{k_{G}},$ representing the ratio of NAPQI forming other protein adducts relative to detoxification by GSH. We then assumed a constant GSH background level to be $gsh_{0},$so that $k_{i}=k_{o}gsh_{0},$ to give,

| $\frac{d\left[ gsh \right]}{dt}=k_{o}gsh_{0}-k_{o}\left[ gsh \right]-\frac{\xi k_{el}\left[ gsh \right]\left[ C_{c} \right]}{\left[ gsh \right]+\frac{k_{p}}{k_{G}}}.$ | (S8) |
| --- | --- |

In summary, the first term in Equation (S8) (and Equation (4) in the main text) relates to the natural production of GSH. The second term in the equation represents the background usage of GSH. The final term in the equation represents the loss of free GSH due to interaction with NAPQI. Equation (S8) is then in the same form as that given in Equation (4) of the main text.

***2.3. Biomarker pharmacodynamic ODE formulation***

Biomarker concentration was plotted against APAP concentration for each biomarker (data not shown) identifying a temporal delay between drug and biomarker accumulation. This hysteresis relationship implies that APAP dynamics regulate a precursor, most probably (GSH), which then influences each biomarker response, and therefore an indirect PD model was chosen to account for this hysteresis delay (10),

|  | $\frac{dr}{dt}=r_{0}k_{out}\left( \frac{R_{50}^{n}+{gsh}_{0}^{n}}{R_{50}^{n}} \right)\left( 1-\frac{{gsh}^{n}}{R_{50}^{n}+gsh^{n}} \right)-k_{out}r.$ | (S9) |
| --- | --- | --- |

In Equation (S9), we assume that the biomarker concentration remains at a baseline steady-state value in the absence of system perturbations. As GSH depletes due to the introduction of APAP, biomarker concentration then increases.

1. **Model Validation**

In order to test the accuracy of the *in-silico* model, data comprising of an experiment carried out at the University of Liverpool consisting of 4 different APAP doses [0, 150, 300, 530] mg/kg and their corresponding biomarker concentrations at 5 h was used for validation (see *model validation – experimental animal treatment* for details). Simulations for identical doses using the *in-silico* model were then computed, and the concentrations at 5 h extracted from the *in-silico* output and compared to the corresponding *in-vivo* mouse data. Both datasets comprised of CD-1 type mice. For GSH, there appears to be an adaptive response in the validation data at low doses which is not included in the *in-silico* model and therefore not portrayed in the simulation However, this is a minor discrepancy and given the large dose range in this validation, the *in-silico* output matches the validation data very well.

| 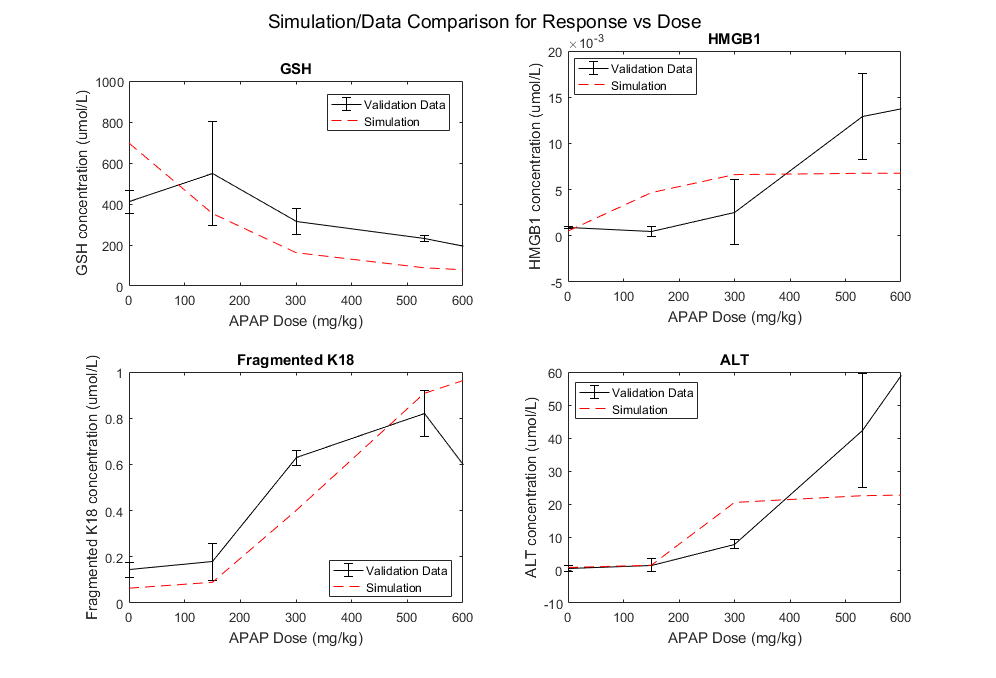 |
| --- |
| **Figure S1:** *In-silico* simulated data versus dose/response validation data used to test the accuracy of the *in-silico* model in new scenarios. |

1. **Statistical Analysis**

**4.1. Creating virtual datasets through *in-silico* simulations**

Each dataset consisted of biomarker concentration time-courses for 1,000 independent and individually distributed *in-silico* mice given a random dose selected from a uniform distribution of range 0-600 mg/kg. Resulting biomarker concentrations were extracted at a random time-point from a uniform range of 0-24 hours. Initial dose amount and time since administration were independent. Since biomarker concentrations had different orders of magnitude, all simulation concentrations were normalised in the range [0,1]. Experimental *(in-vitro)* noise (e.g. unexplained PK variability following i.p. administration in the mouse) was mimicked in the *in-silico* data set by applying *in-vitro* observed standard deviations in biomarker concentrations from an APAP study performed by Antoine *et al.* (2009) (ALT = 11.22, HMGB1 = 0.00097, K18 = 2.39, Fragmented K18 = 0.12 μmol/l).

**4.2. Justification for choice of robust multiple linear regression model for predicting time since administration and initial dose as continuous variables**

Normality tests indicated that the simulated data was non-normally distributed (11). Improvements to the linear model, made by employing interaction/polynomic terms, were insignificant and hence a robust multiple linear regression model was chosen to predict time since administration and initial dose as continuous variables.

**4.3 Visualisation**

To examine class structure and separability whilst retaining model variation, we applied Principal Component Analysis (PCA) and constructed two-dimensional scatter plots of the in-silico derived data projected onto the ﬁrst two principal components. Additionally, to visually expose class structure, a fast tree-based implementation of the T-SNE method was employed (12).

***4.4. Classification techniques used to classify time since administration and initial dose***

For classifying time-since-administration, the critical ranges were defined as (0-2], (2-5], (5-10], (10-15] and (15-24] hours, complying with the current liver histology phases standard. For dose, the ranges were [0-200], [201-400] and [401-600] mg/kg, capturing therapeutic, small, and large (overdoses) respectively.

Multinomial logistic regression (13) was used as a method that fits well when multiple response categories are available. Since response categories were in an ordinal manner, ordinal multinomial logistic regression was also used. Both linear and quadratic discriminant analysis were employed (14). A naïve Bayes classifier (15) was also used to predict class probability. Additional model-free classification techniques k nearest neighbour (k-nn) and optimal weighted nearest neighbour were also employed (16,17) to test for robustness; since classes are not previously defined for these methods, observations group together based solely on their similarity, and the aim would be to determine whether similar observations automatically group into our desirable classes.

***4.5 Predicting the probability of liver injury***

The histology scores were binarised where any mouse that portrayed normal/minimal necrosis (score 0/1) was mapped to 0 (representing no liver injury) whilst moderate/severe necrosis (score 2/3) was mapped to 1 (representing liver injury occurrence). Binary logistic regression analysis (18) was used to predict DILI since the outcome variable (histology score) was now dichotomous, i.e. liver injury or no liver injury.

***4.5.1 Formulae for logistic regression model outputs***

Upon applying a binary logistic regression analysis in SPSS statistical software, the output was a logit equation based solely on HMGB1 concentration,

|  | $L_{eq} =0.635*\left( HMGB1 concentration \right)-3.870.$ | (S10) |
| --- | --- | --- |

This equation was substituted into a standard probability equation, resulting in the final liver damage probability equation used in our analysis,

|  | $Probability =\frac{1}{1+e^{-L_{eq}}}.$ | (S11) |
| --- | --- | --- |

1. **Visualising the probability of liver injury following an APAP dose**

As described in the main article, Principal Component Analysis (PCA) and T-SNE methods were used to visualise 1000 *in-silico* observations with regards to initial dose amount and time since ingestion based on a combination of biomarker concentrations (see Figure 2, main article). In this analysis, a dose and time category can be determined for each observation. This then defines a sub-region of dose/time parameter space within our liver damage predictions (Figure 3G). We can obtain the mean and maximum probabilities of liver injury from this sub-region and then map this back onto the original observation point on the PCA/TSNE visualisations to predict liver injury probability.

| 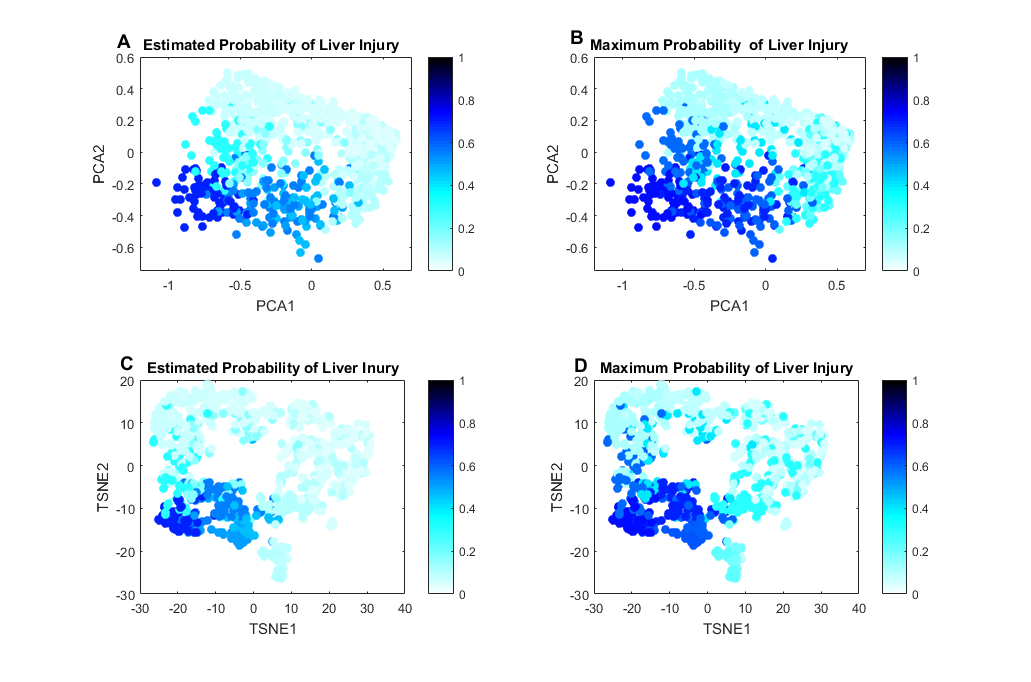 |
| --- |
| **Figure S2:** Visualising the probability of liver injury for each of the *in-silico*-derived observations. For both metrics (mean and max), observations with a high probability of liver injury are clearly clustered within the parameter space and separable from low probability cases, with the T-SNE method showing better separation. |

1. **Sensitivity Analysis**

We calculated time-dependent sensitivities of all model variables (APAP, GSH, ALT, HMGB1, Full K18 and Fragmented K18 concentrations) with respect to all model parameters using the Mathworks’ Simbiology toolbox (1). If we define model output as $x(t)$ and two model parameters as $y$and $z$, the time-dependent sensitivities of output $x$ with respect to each parameter value are the scaled time-dependent derivatives,

$$\frac{{\partial x}/x}{{\partial y}/y},\frac{{\partial x}/x}{{\partial z}/z},$$

where the numerator is the sensitivity output and the denominators are sensitivity inputs to the analysis.

| 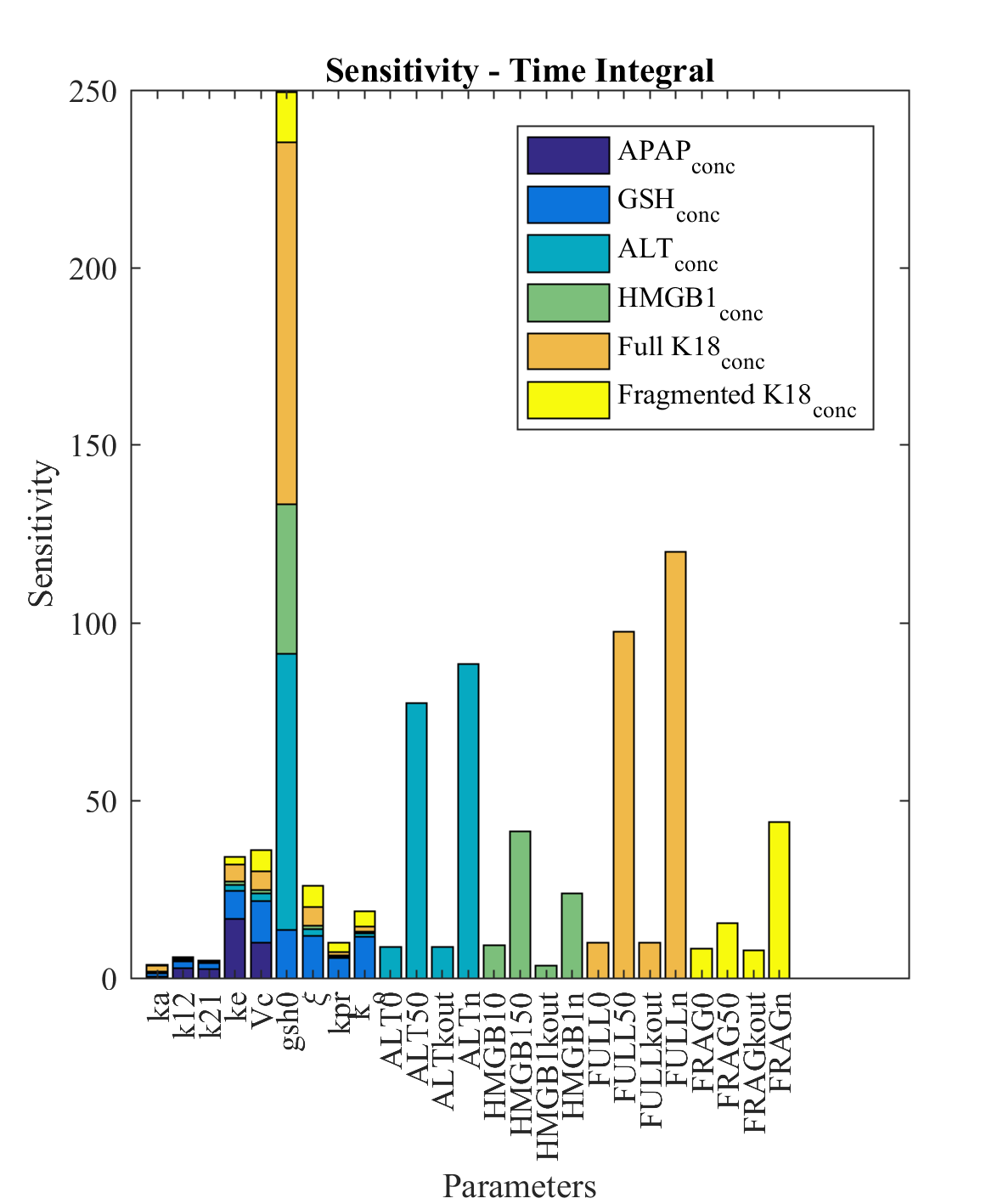 |
| --- |
| **Figure S3:** Sensitivity analysis of the *in-silico* model parameters – visualising the change in model output with regard to perturbations in model parameters. |

The results of the analysis in Figure S3 portray the level of sensitivity each of the *in-silico* outputs has with regard to perturbations in each of the model parameters. With reference to table S1, we observe that any parameters deemed to be sensitive by this analysis also have small standard errors for the parameter estimates which gives us confidence in the robustness of these predicted values. Although some parameters have greater standard-error estimates e.g. $k_{pr}$, and $k_{o}$ from the GSH model, the sensitivity analysis shows that these are not highly sensitive and therefore impact minimally on the outputs of the model.

Importantly, this sensitivity analysis allows us to identify the most sensitive parameters which would potentially require most attention if translating this model to a human clinical Pop-PK framework. As we can see from Figure S3, the most sensitive parameter is the baseline level of GSH, $gsh_{0}$. Since this value is based on experimental estimates in this study (4), we are confident that the results are robust for the mouse situation. However, this parameter is clearly of importance for the predictivity of this model structure so will need to be of particular focus when translating to the clinical case. In addition to this parameter, the elimination rate, $k_{el}$ and the volume of the central compartment, $V_{c}$ are also highlighted as being sensitive and so they should therefore be given special consideration when translating into the clinical context. For the PD element of the model, the proportion of CYP-activated APAP which is transformed into NAPQI, $\xi$, is also deemed sensitive. Since this quantity will be dependent on baseline GSH this result is not particularly surprising, and the importance of further investigation into this parameter has already been highlighted. Biomarkers ALT and Full K18 are identified as being sensitive to parameter changes, highlighting the need for sufficiently dense data for these biomarkers if they were to be used in a translated human model. Biomarkers HMGB1 and Fragmented K18 are identified as being relatively less sensitive and more robust to parameter changes.

**References**

1. Mathworks. Simbiology Toolbox: User’s guide (R2017b). Natick, Massachusetts: The MathWorks Inc.; 2016.

2. Coen M, Ruepp SU, Lindon JC, Nicholson JK, Pognan F, Lenz EM, et al. Integrated application of transcriptomics and metabonomics yields new insight into the toxicity due to paracetamol in the mouse. J Pharm Biomed Anal. 2004;35(1):93–105.

3. Antoine DJ, Williams DP, Jenkins AK, Regan SL, Sathish JG, Kitteringham NR, et al. High-mobility group box-1 protein and keratin-18, circulating serum proteins informative of acetaminophen-induced necrosis and apoptosis in vivo. Toxicol Sci. 2009;112(2):521–31.

4. Antoine DJ. Chemical and molecular markers of hepatic drug bioactivation, apoptosis and necrosis [Internet]. University of Liverpool; 2009. Available from: http://ethos.bl.uk/OrderDetails.do?uin=uk.bl.ethos.501593

5. Dhillon S, Gill K. Basic Pharmacokinetics. In: Clinical Pharmaokinetics [Internet]. 2006 [cited 2017 May 12]. p. 1–44. Available from: http://www.dandybooksellers.com/acatalog/9780853695714.pdf

6. Athena Z, Gregory H, Jeffrey B, Brian K, Nastya K, Samer M, et al. Safety and Population Pharmacokinetic Analysis of Intraveneous Acetaminophen in Neonates, Infants, Children and Adolescents with pain or fever. J Pediatr Pharmacol Ther. 2014;17(4):507–18.

7. Mohammed B, Engelhardt T, Cameron G, Cameron L, Hawksworth G, Hawwa A, et al. Population pharmacokinetics of single-dose intravenous paracetamol in children. Br J Anaesth. 2012;108(5):823–9.

8. Hinson JA, Roberts DW, James LP. Mechanisms of Acetaminophen-Induced Liver Necrosis. Pharmacology [Internet]. 2010;196(196):1–34. Available from: http://www.springerlink.com/index/10.1007/978-3-642-00663-0

9. Prescott L. Kinetics and metabolism of paracetamol and phenacetin. Br J Clin Pharmacol. 1980;10(2 S):291S–298S.

10. Teuscher N. What are direct and indirect pharmacodynamic models? 2011.

11. Franc J. Robust regression - Robust estimation of regression coefficients in linear regression model when orthogonality condition is breaking. 2017. p. 1–45.

12. Maaten L Van Der. Accelerating t-SNE using tree-based algorithms. 2014;15:3221–45.

13. Starkweather J, Moske AK. Multinomial logistic regression. Multinomial Logist Regres. 2011;51(6):404–10.

14. Welling M. Fisher Linear Discriminant Analysis. Science (80- ) [Internet]. 2009;1(2):1–3. Available from: http://www.cs.huji.ac.il/~csip/Fisher-LDA.pdf

15. Leung KM. Naive bayesian classifier. Polytech Univ Dep Comput Sci Risk Eng. 2007;

16. Kuhkan M. A Method to Improve the Accuracy of K-Nearest Neighbor Algorithm. Int J Comput Eng Inf Technol (IJCEIT), Vol 8, Issue 6, June 2016 [Internet]. 2016 [cited 2017 May 9];8(6):90–5. Available from: http://www.ijceit.org/published/volume8/issue6/1Vol8No6.pdf

17. Samworth RJ. OPTIMAL WEIGHTED NEAREST NEIGHBOUR CLASSIFIERS 1. Ann Stat [Internet]. 2012 [cited 2017 May 9];40(5):2733–63. Available from: http://www.statslab.cam.ac.uk/~rjs57/AOS1049.pdf

18. Wuensch K. Binary Logistic Regression with SPSS. 2016.
